# Progressive pulmonary fibrosis: the importance of identification and intervention

**DOI:** 10.1183/16000617.0051-2025

**Published:** 2026-03-25

**Authors:** Philip L. Molyneaux, Toby M. Maher

**Affiliations:** 1 Imperial College London, London, UK; 2 Keck School of Medicine, University of Southern California, Los Angeles, CA, USA

## Abstract

The concept of progressive pulmonary fibrosis (PPF) was developed to facilitate the identification of patients with an interstitial lung disease (ILD) that is worsening and requires treatment. Various criteria have been proposed to identify PPF, generally based on a deterioration in forced vital capacity alone or with worsening of respiratory symptoms and/or radiological abnormalities. All these criteria are imperfect and based on a limited evidence base. PPF, however it is defined, is associated with high morbidity and mortality. In clinical practice, flexibility is needed in defining ILD progression given differences in the frequencies and methodologies used to monitor patients’ disease. Prompt identification of PPF is important to enable timely initiation or escalation of treatment to slow progression of lung fibrosis, consider eligibility for lung transplantation and provide supportive care as needed. In future, earlier treatment of patients at risk of progression may be possible to improve outcomes for patients.

## Shareable abstract

**Prompt identification of disease progression in patients with interstitial lung disease is important so that initiation or escalation of treatment can be considered and supportive care provided as needed.** https://bit.ly/4os4zCo

## Introduction

The concept of progressive fibrosing interstitial lung disease (PF-ILD) or progressive pulmonary fibrosis (PPF) was developed to facilitate the identification of patients with an ILD other than idiopathic pulmonary fibrosis (IPF) that is worsening and requires treatment [1, 2]. This construct has allowed the clinical development of therapies for these patients [3, 4]. Varying definitions of PPF have been used in clinical trials [3–7] and observational studies [8–10] or been proposed by respiratory societies [11] and other panels of experts in the field [12–14]. These criteria are generally based on a minimum observed deterioration in forced vital capacity (FVC) alone or together with worsening of respiratory symptoms and/or radiological abnormalities. PPF is associated with poor outcomes, including high mortality [9, 15–17]. In this article, we discuss the importance of early identification and treatment of PPF. For the sake of simplicity, we use the term PPF throughout this review to mean progressive fibrotic ILD irrespective of the specific criteria used to define progression.

## Definitions of PPF

**FIGURE 1:**
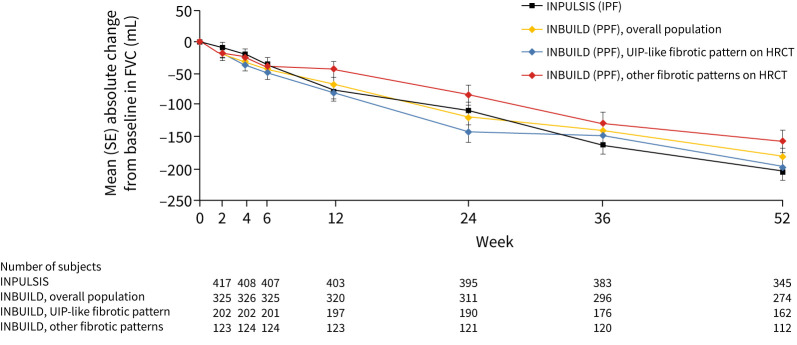
Changes in forced vital capacity (FVC) in the placebo groups of the INPULSIS trials in patients with idiopathic pulmonary fibrosis (IPF) and the INBUILD trial in patients with progressive pulmonary fibrosis (PPF). HRCT: high-resolution computed tomography; UIP: usual interstitial pneumonia. Reproduced from [18] with permission.

The concept of PPF started being widely accepted following publication of the results of the INBUILD trial of nintedanib, which showed, for the first time, the efficacy of a drug in slowing the progression of fibrotic ILDs other than IPF [3]. The eligibility criteria used in the INBUILD trial (table 1) were based on parameters commonly assessed in the management of patients with ILDs, occurring at any time within the prior 2 years despite management of the ILD as deemed appropriate in clinical practice [3]. A striking observation, given that IPF is known to be associated with very high morbidity and mortality, was that the decline in FVC over 1 year in the placebo group of the INBUILD trial was similar to the decline observed in the placebo arms of the INPULSIS trials in patients with IPF (−193 mL·year^−1^ and −221 mL·year^−1^, respectively) [18]. Additionally, while the decline in FVC in patients with PPF in the INBUILD trial was greater in those with a usual interstitial pneumonia (UIP) pattern on high-resolution computed tomography (HRCT), patients with other fibrotic patterns on computed tomography (CT) still had a substantial decline in FVC over 1 year (figure 1). Declines in FVC were similar irrespective of the underlying ILD leading to PPF [18].

**TABLE 1:** Criteria for identification of progressive pulmonary fibrosis (PPF) used in clinical trials and proposed in the American Thoracic Society (ATS)/European Respiratory Society (ERS)/Japanese Respiratory Society) (JRS)/Latin American Thoracic Association (ALAT) clinical practice guidelines

Trial or guidelines	Criteria
**INBUILD trial of nintedanib [3]**	≥1 of the following at any time within 2 years, despite management deemed appropriate in clinical practice: 1) Relative decline in FVC % pred ≥10% 2) Relative decline in FVC % pred ≥5–<10% and increased extent of fibrosis on HRCT 3) Relative decline in FVC % pred ≥5–<10% and worsened respiratory symptoms 4) Worsened respiratory symptoms and increased extent of fibrosis on HRCT
**RELIEF trial of pirfenidone in PPF [6]**	Annual decline in FVC % pred ≥5%, based on ≥3 FVC measurements within 6–24 months, despite conventional therapy
**Trial of pirfenidone in unclassifiable PPF [5]**	≥1 of the following within 6 months: 1) Absolute decline in FVC % pred >5% 2) Worsening symptoms
**ATS/ERS/JRS/ALAT clinical practice guidelines [11]**	≥2 of the following within 1 year: 1) Worsening respiratory symptoms 2) Physiological evidence of disease progression, defined as either: a) Absolute decline in FVC % pred >5% b) Absolute decline in *D*_LCO_ % pred >10% 3) Radiological evidence of disease progression, defined as ≥1 of the following: a) Increased extent or severity of traction bronchiectasis and bronchiolectasis b) New ground-glass opacity with traction bronchiectasis c) New fine reticulation d) Increased extent or increased coarseness of reticular abnormality e) New or increased honeycombing f) Increased lobar volume loss

*D*_LCO_: diffusing capacity of the lung for carbon monoxide; FVC: forced vital capacity; HRCT: high-resolution computed tomography.

The term PPF was coined in guidelines published by the US, European, Japanese and Latin American respiratory societies in May 2022 [11]. The criteria for PPF proposed in this guideline were based on similar factors to those used as eligibility criteria in the INBUILD trial, though with different thresholds and combinations of parameters. Further, the guidelines suggested that only change in the prior 1 year should be used to determine progression (table 1). The different criteria used in the INBUILD trial and proposed by the international guidelines have generated an element of confusion, with some people believing there is an entity called “PF-ILD” defined based on the INBUILD trial criteria and an entity called “PPF” defined by guideline criteria. In fact, we believe that these criteria (and others) simply represent differing approaches to addressing the same clinical challenge: how to identify individuals with an ILD that is actively worsening and thus causing morbidity and a greater risk of mortality. All such criteria are imperfect and based on a limited evidence base. Reflecting the current uncertainties, a recent Delphi exercise demonstrated a limited consensus among physicians regarding how PPF should be defined [14].

An under-recognised challenge of identifying ILD progression is that while physiological parameters (*e.g.,* FVC) are measured on a reproducible scale that permits precise assessment of change, the same is not true for symptoms or abnormalities on CT imaging. Both the international guideline and the INBUILD trial criteria simply required change in symptoms and/or CT appearances without defining the magnitude of change, or on what scale it should be measured [3, 11]. The tools used to assess symptoms in patients with PPF have limitations. Most were not developed in patients with pulmonary fibrosis. All require further validation and the determination of minimal clinically important differences using data from independent cohorts. The Living with Pulmonary Fibrosis (L-PF) questionnaire, developed in patients with IPF [19], showed promise as a measure of changes in dyspnoea and cough in the INBUILD trial in patients with PPF, in which a benefit of nintedanib was demonstrated [20]. However, this tool did not show a benefit of nerandomilast on respiratory symptoms in the FIBRONEER-ILD trial in patients with PPF, despite benefits of therapy on FVC decline and the initiation of supplemental oxygen [4]. It is possible that simple measures of symptoms, such as single questions answered on a visual analogue scale, may have advantages over more complex questionnaires, but more research is needed.

Visual assessment of fibrotic features is limited by high inter-observer variability and poor ability to detect change [21, 22]. Ongoing research and the introduction of computerised quantitative assessment of CT images may provide more reproducible measures of the extent of radiological abnormalities and enable detection of small changes [23, 24]. However, such tools require further validation before they can be implemented into clinical practice.

## Pathogenesis of PPF

The pathogenesis of pulmonary fibrosis involves a complex interplay among genetics, cellular senescence and inflammatory and fibrotic pathways. In susceptible individuals, repeated injury of alveolar epithelial cells promotes recruitment and activation of fibroblasts [25]. The release of profibrotic growth factors and cytokines induces further proliferation and activation of fibroblasts and their differentiation to myofibroblasts, leading to excess deposition of extracellular matrix (ECM) proteins such as collagen and stiffening of the ECM [25, 26]. These processes induce further activation and differentiation of fibroblasts, creating a feed-forward loop that drives further fibrosis [27, 28]. Through these mechanisms, fibrotic pathways become self-sustaining, leading to inexorable progression of the fibrosis irrespective of the initial injury. There appears to be considerable overlap in the mechanisms that drive disease progression in individuals with IPF and in those with PPF associated with other ILDs [29].

## Outcomes in patients with PPF

**FIGURE 2:**
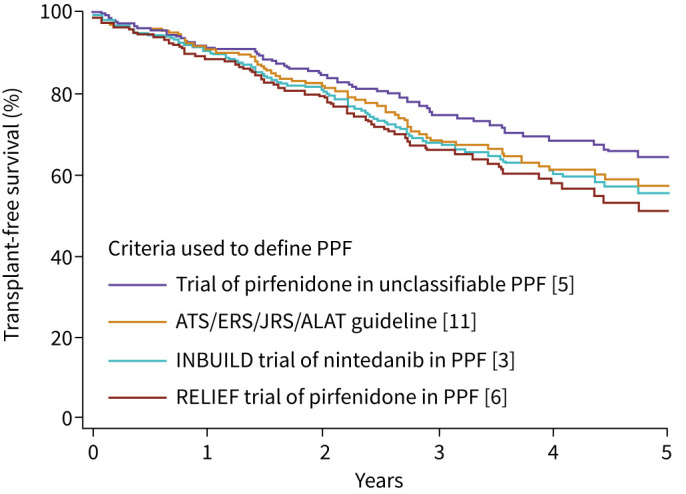
Transplant-free survival in 753 patients with progressive pulmonary fibrosis (PPF) enrolled in the Austin Health ILD Registry or Canadian Registry for Pulmonary Fibrosis. Reproduced from [16] with permission. ALAT: Latin American Thoracic Association; ATS: American Thoracic Society; ERS: European Respiratory Society; JRS: Japanese Respiratory Society.

**FIGURE 3:**
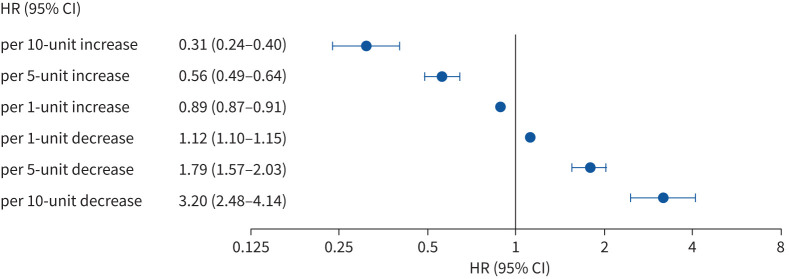
Associations between the rate of change in forced vital capacity (FVC) % predicted and risk of death over 52 weeks in clinical trials of nintedanib in 2553 patients with pulmonary fibrosis. Data from [40]. HR: hazard ratio.

Several studies have investigated short-term changes in FVC and mortality in patients who meet different criteria for PPF [9, 16, 17, 30–38]. An analysis of retrospective data from 1227 patients at four centres, which assessed 1-year decline in FVC after a patient had fulfilled one of nine criteria for progression, showed that all the criteria were associated with decline in FVC over the following year, but there was substantial heterogeneity based on the criteria used and the underlying ILD [32]. Irrespective of the criteria used, prior progression of fibrotic ILD is associated with an increased risk of mortality [16] (figure 2). In a retrospective, multicentre cohort study, the performance characteristics of eight lung function-based measures of PPF and the guideline criteria for PPF for discriminating death or lung transplant in the following 2 years were assessed [38]. The results showed that a relative decline in FVC ≥10% over 12 months, a relative decline in diffusing capacity of the lung for carbon monoxide (*D*_LCO_) ≥15% over 12 months and the guideline criteria for PPF most accurately predicted death or transplant [38]. Retrospective data from 1341 patients at four centres showed that 11 of 14 criteria for PPF were associated with an increased risk of death or lung transplant in the following 5 years, with even “small” declines in FVC associated with increased mortality [17]. An increased risk of mortality associated with “small” (*i.e.,* <10%) declines in FVC has also been demonstrated in other studies in patients with IPF and other ILDs (figure 3) [39–41]. “Small” degrees of worsening in radiological abnormalities assessed using quantitative CT have also been associated with poor outcome [42–45]. Studies in patients with various fibrotic ILDs have shown a relationship between short-term worsening of dyspnoea and mortality [46–48].

## Clinical practice

In our opinion, identifying the specific criteria that should be used to define PPF is less important than recognising that patients who have any signs of worsening ILD have active disease, a poor prognosis, and may benefit from treatment. Pulmonary fibrosis leads to irreversible destruction of alveolar tissue with worsening of symptoms and quality of life. Prompt identification of PPF is therefore important to enable timely initiation of treatment. In clinical practice, flexibility is needed in defining ILD progression due to differences in the frequencies and methodologies used to monitor patients [49–52]. Applying strict criteria for progression, irrespective of when investigations such as pulmonary function tests (PFTs) and CT scans are conducted, may result in patients with progressive disease being incorrectly labelled as having stable disease. Patients understand the importance of PFTs and CT scans for monitoring progression of their disease, despite uncertainties in their interpretation [53, 54].

The term PPF may be used to describe progressive lung fibrosis *per se* or progressive lung fibrosis in a patient who has received therapy for their ILD and is progressing despite treatment. The latter has been referred to as progression despite “conventional therapy” [6] or even despite “optimal therapy” [55], but in reality, for the vast majority of ILDs, there is no standard of care or the treatments used are not supported by a meaningful evidence base. This creates a challenge in defining PPF as only occurring in patients who have received “standard management” or in only using nintedanib (the only drug licensed for the treatment of PPF) to treat PPF in patients who have “failed standard management” and may lead to a delay in patients receiving a treatment that slows the progression of their disease.

Patients with ILD need an accurate diagnosis and should receive first-line treatment appropriate to their ILD [56]. For patients with IPF, this should be an approved antifibrotic therapy (nintedanib or pirfenidone) [57]. For patients with ILD due to a connective tissue disease (CTD), immunosuppressant therapy may be efficacious in improving lung function, although the evidence base is weak for diseases other than systemic sclerosis [58, 59]. In our opinion, antifibrotic therapy should be considered for treatment of CTD-ILDs, even if the patient is taking immunosuppressant therapy to treat the systemic disease, in the same way that use of immunosuppressant therapy does not negate the need to treat other organ-specific complications such as renal or cardiac involvement or pulmonary hypertension. For patients with ILDs unrelated to CTDs, there is no evidence-based treatment for patients who do not meet criteria for PPF and the treatments used in practice are highly variable. In our opinion, consideration should be given to using antifibrotic therapy as first-line treatment in patients in whom fibrosis is the predominant feature [60], including those with fibrotic hypersensitivity pneumonitis, fibrotic idiopathic nonspecific interstitial pneumonia (NSIP) and unclassifiable ILD, which is associated with particularly high mortality [61, 62]. An NSIP pattern on HRCT should not be assumed to reflect inflammatory disease, as NSIP is most frequently fibrotic and progressive [63, 64].

Therapeutic decisions should be made on a case-by-case basis, taking into account evidence of progression, risk factors for progression and the patient's preferences. A proportion of patients with ILDs will respond well to treatment of the underlying ILD (*e.g.* antigen avoidance in hypersensitivity pneumonitis, immunomodulatory therapy of systemic autoimmune rheumatic diseases) and never require antifibrotic therapy. Irrespective of treatment, patients with fibrotic ILDs should be monitored carefully for progression [11, 13, 14, 65, 66]. The frequency of monitoring should be individualised based on disease severity, risk factors for progression, and the underlying disease [66]. Identification of PPF should prompt consideration of initiation or escalation of treatment with a view to slowing further progression [11, 13, 58]. However, patients with PPF will generally continue to progress even when treated with effective therapies [4, 67, 68]. Interventions to relieve symptoms and preserve quality of life, which may include pulmonary rehabilitation, light exercise or breathing techniques, as well as pharmacological therapies and supplemental oxygen, should be provided as appropriate [69–74]. Advance care planning and the provision of palliative care should also be considered. Evaluation for lung transplant should be discussed at an early stage with patients who may be candidates [75]. Importantly, throughout the patient journey, the patient's preferences should be taken into account in how they are monitored and treated, ensuring that patients understand the risks and benefits of available treatments and of declining treatment [54, 74]. Physicians may value training in how to communicate effectively with patients with IPF/PPF to provide more empathetic care and enable shared decision-making [76].

## Future directions

PPF remains an active area of clinical research, with several phase II/III trials of new therapies recruiting or in progress (table 2). However, once PPF has developed, the patient has already entered a self-sustaining phase of fibrotic disease that will ultimately be life-shortening [17, 28]. Although PPF can be treated, it is unlikely that any drug regimen will succeed in halting the progression of pulmonary fibrosis once it has reached a late stage. What is needed are means of identifying patients with early ILD, or even individuals with fibrotic interstitial lung abnormalities, who are at risk of progression and for whom early provision of treatment could prevent progression in the long term [77–79]. Physiological predictors of PPF include older age, male sex, lower FVC and lower *D*_LCO_ [80], but it seems likely that genetic, proteomic and/or radiological biomarkers will be required to identify patients at risk of fibrotic progression. Mutations in telomere-related genes and shortened telomeres have been associated with an increased risk of disease progression in patients with pulmonary fibrosis [38, 81, 82], while a 52-gene signature has been shown to be a predictor of transplant-free survival in patients with IPF [83–85]. In future, circulating protein biomarkers may enable management strategies to be based on more accurate prediction of disease progression and likely response to particular therapies [74, 86–88]. The use of quantitative measures of the extent of fibrosis and other radiological abnormalities may enable earlier and more accurate identification of progression [45, 89, 90]. The availability of drugs with tolerability profiles that make it feasible for them to be used in patients at an early stage of ILD will be a prerequisite for delivering therapy aimed at preventing progression.

**TABLE 2:** Ongoing phase II/III trials in patients with progressive pulmonary fibrosis (PPF)

Trial name/identifier	ALOFT-PPF (NCT06025578)	TETON-PPF (NCT05943535)	NCT06329401	NCT05139719
**Phase**	III	III	IIb	IIb
**Treatment**	Admilparant 60 mg or 120 mg or placebo twice daily	Treprostinil inhalation solution or placebo four times daily	Low- or high-dose AP01 pirfenidone solution for inhalation or placebo twice daily	HEC585 (a pyrimidone analogue derived from pirfenidone) or placebo once daily
**Key inclusion criteria**	PPF Extent of fibrosis on HRCT ≥10% FVC ≥40% pred Nintedanib or pirfenidone permitted if stable for ≥90 days Immunosuppressive therapy permitted if stable for ≥90 days	PPF Extent of fibrosis on HRCT >10% FVC ≥45% pred Nintedanib or pirfenidone permitted if stable for ≥90 days Immunosuppressive therapy permitted if stable for ≥120 days	PPF Nintedanib permitted if stable for ≥6 months	PPF Extent of fibrosis on HRCT >10% FVC ≥45% pred
**Estimated number of patients**	1092	698	300	110
**Primary end-point(s)**	Frequency of spontaneous syncopal events at ∼4 weeks (cohort 1) Absolute change in FVC (mL) at week 52 (cohort 2)	Absolute change in FVC (mL) at week 52	Absolute change in FVC (mL) at week 52	Absolute change in FVC (mL) at week 24

FVC: forced vital capacity; HRCT: high-resolution computed tomography.

## Conclusions

PPF, however defined, is associated with high morbidity and mortality. Patients with fibrosing ILDs need careful monitoring for progression. Detection of PPF should prompt consideration of change in therapy and the provision of supportive care as needed. Identification of patients with progressive disease at an early stage or even prior to progression would enable earlier initiation of treatment to improve outcomes.

**Points for clinical practice**

- Patients with ILD should receive first-line treatment appropriate to their disease.
- Patients with fibrosing ILDs need regular monitoring for progression.
- Various criteria may be used to identify PPF. However it is defined, PPF is a sign of active disease and is associated with poor outcomes.
- Identification of progression should prompt consideration of initiation or escalation of treatment to slow disease progression.
- Patients with PPF should be offered supportive care as needed.

## Supplementary material

**Please note:** supplementary material is not edited by the Editorial Office, and is uploaded as it has been supplied by the author. Supplementary podcast ERR-0051-2025.SUPPLEMENT Supplementary podcast transcript ERR-0051-2025.SUPPLEMENT
