# Supplementary material for: Progressive pulmonary fibrosis: the importance of identification and intervention
Source: Eur Respir Rev. 2026 Mar 25;35(179):250051. doi: 10.1183/16000617.0051-2025 (PMC13014284; doi:10.1183/16000617.0051-2025)
Supplement: Supplementary file 2 [file ERR-0051-2025.SUPPLEMENT.pdf]

Transcript of podcast on identification of PPF

AI voice: *This podcast with Professor Toby Maher and Professor Philip Molyneaux has been created as an extender to the article entitled "Progressive pulmonary fibrosis: the importance of identification and intervention" published in the European Respiratory Review.*

Toby: Hi, I'm Toby Maher, I'm a pulmonologist from the Keck School of Medicine at the University of Southern California in Los Angeles.

Phil: Hi, I'm Phil Molyneaux, a pulmonologist from Imperial College London in the UK.

Toby: Today we're going to talk about progressive pulmonary fibrosis, or PPF, and why it's so important that PPF is identified and treated. PPF is a term used to describe worsening pulmonary fibrosis in patients who have an interstitial lung disease other than idiopathic pulmonary fibrosis, or IPF. IPF is not included in the definition of PPF, as it's inherently progressive. PPF is also referred to as progressive fibrosing interstitial lung disease, or PF-ILD.

Phil: Yes, the terms can get a bit confusing here. The term PF-ILD was used in the INBUILD trial of nintedanib: this was the first trial to look at the effects of antifibrotic drugs in patients with non-idiopathic progressive fibrotic ILD. Then the term PPF was coined in a clinical practice guidelines published in 2022. The criteria used to define PPF in the guideline were different to those used in the INBUILD trial, but both included measures of lung function decline, worsening fibrosis on CT, and worsening respiratory symptoms. Other sets of criteria to define PPF have also been proposed, but what's really important is that however progression of ILD is defined, it's associated with very poor outcomes, including high mortality.

Toby: That's right. The different criteria that have been proposed represent differing approaches to addressing the same clinical challenge, *i.e.* how to identify ILD that is worsening and causing morbidity and premature death. All the criteria proposed are imperfect and based on limited evidence, but for patients with PPF, the important thing is that they are identified and treated.

Phil: Yes, it's worth remembering that the progression of pulmonary fibrosis involves a self-sustaining process of lung damage. Once PPF has developed, progressive destruction of lung tissue results in decline in lung function, worsening symptoms, and a poorer prognosis. It's important that PPF's identified as soon as possible, so that treatment can be started to slow progression and improve outcomes. Physicians need to know how to recognise progression of ILD and monitor their patients for sign of progression, so they can call PPF when they see it.

Toby: Yes, rather than applying strict thresholds for degrees of worsening, or the time period over which worsening has to happen, the important thing is to recognize progression promptly and intervene to slow further damage. This is another area in which the terms used can be confusing. The term PPF may be used to describe progressive lung fibrosis *per se* or progression despite treatment. The challenge we have is that for the majority of ILDs, there is no standard of care, and most of the drugs that are used are not supported by an adequate evidence base. We need to make sure that when PPF is identified, we do not delay starting a treatment that has been proven to slow progression of PPF.

Phil: Yes, prompt intervention is important. For some patients, this will involve starting treatment and for others, escalating existing therapy. A patient with ILD due to an autoimmune disease will likely be taking immunosuppressive therapy already. This may have some benefits on lung function, but doesn't mean they can't also be given therapy specifically to treat their PPF. This is one example of where a multidisciplinary approach, involving input from both a pulmonologist and rheumatologist, can be really valuable. Patients may also have PPF secondary to other diseases, such as hypersensitivity pneumonitis or unclassifiable interstitial lung disease. Treatment decisions need to be made on a case-by-case basis, but if a patient has progressive lung fibrosis, they should be offered a treatment to slow its progression and they should continue to be monitored.

Toby: Yes, we need to take PPF seriously, as without treatment, patients' outcomes are very poor. And it's important to remember that as well as therapies to slow progression, patients with PPF may benefit from medications to help alleviate their symptoms or to treat comorbidities, as well as pulmonary rehabilitation, oxygen therapy, or evaluation for lung transplant. Advance care planning should also be considered. Decisions around treatment should be individualised, taking into account what the patient wants. Helping patients understand the risk and benefits of available treatments is key to enable them to participate in decisions about their care.

Phil: Absolutely. Looking to the future, PPF remains a field of active research, with new drugs being developed and more studies into predictors of PPF. If we could identify patients with early ILD who are likely to develop PPF, we might be able to intervene early to delay PPF starting. That would likely improve outcomes to a much greater extent than only treating patients who already have PPF.

Toby: Indeed. We have high hopes for the future. Thanks, Phil, this has been a great conversation.

Phil: Thank you, Toby, and thank you for listening for this podcast.

AI voice: *This podcast is an extender to an article published in the European Respiratory Review. The authors meet criteria for authorship as recommended by the International Committee of Medical Journal Editors. The authors did not receive payment related to the development of the article or podcast. Elizabeth Ng and Wendy Morris of Fleishman-Hillard, London, UK provided editorial support, which was contracted and funded by Boehringer Ingelheim. Boehringer Ingelheim was given the opportunity to review the article and podcast for medical and scientific accuracy, as well as intellectual property considerations.*
